# Multinational physician perspectives on abortion care in the context of changing legislation

**DOI:** 10.3389/fgwh.2025.1581704

**Published:** 2025-11-27

**Authors:** Emilie S. Allaert, Caroline C. Liu, Katherine Liu, Anna T. Truong, Michael S. Wilkes

**Affiliations:** 1 School of Medicine, University of California Davis, Sacramento, CA, United States; 2 Office of the Dean, School of Medicine, University of California Davis, Sacramento, CA, United States

**Keywords:** reproductive health policy, abortion care, access to abortion care, Dobbs v. Jackson, Roe v. Wade, eighth amendment, abortion act 1967, human life protection act

## Abstract

**Introduction:**

*Since Dobbs v. Jackson Women's Health Organization* (2022) overturned *Roe v. Wade* (1973), thus returning abortion policy decisions to state governments, abortion access across the United States became fragmented, with some states enacting near-total bans and other states strengthening protection. As a parallel, the Republic of Ireland's (ROI) 2018 repeal of the Eighth Amendment and the United Kingdom's (UK) longstanding framework of care offer informative historical examples. This qualitative study explores the perspectives and experiences of abortion-trained physicians in California (CA), Texas (TX), ROI, and the UK, focusing on how legislation shapes physicians' ability to deliver comprehensive abortion care.

**Methods:**

In accordance with Consolidated Criteria for Reporting Qualitative Research (COREQ), nineteen abortion-trained physicians practicing in Family Medicine, Obstetrics & Gynecology, and General Practice (CA *n* = 6; TX *n* = 4; UK *n* = 4; ROI *n* = 5) participated in 1-hour semi-structured interviews from August 2022 to November 2023 relating to their abortion care training and practice. Interviews were audio-recorded, transcribed, anonymized, and coded using Braun and Clarke's six-step approach to thematic analysis and conducted until thematic saturation was reached.

**Results:**

Analysis revealed several interconnected themes. Across all geographical practices, physicians highlighted the importance of centering care on patients' needs, but variations in legislation largely shaped clinical care. Training experience varied widely with many shaping their own education in the context of available resources. Changing policies functioned as a clinical variable, often shifting with cultural and political attitudes. Geographic, financial, facility-related, and healthcare infrastructure barriers compounded legislative obstacles, highlighting that legality does not guarantee accessibility. Participants additionally emphasized cross-specialty advocacy, reported experiences with stigma, and dispelled common misconceptions on abortion.

**Discussion:**

These findings highlight that policy functions as a major determinant of health and that centering on patient experiences, standardizing education, addressing healthcare infrastructure barriers, strengthening peer support systems, continued physician advocacy, and systemic reforms are necessary to reduce preventable delays, patient distress, and disparities in care. This study highlights the importance of incorporating physicians' perspectives into legislative discussions to ensure accurate representation of patient needs and challenges in accessing abortion care.

## Introduction

Across the world, abortion regulations in recent years have trended towards liberalization (1). In one such country, the Republic of Ireland (ROI), abortion laws have undergone significant changes, initially stringent with the 1861 Offenses Against the Person Act and further solidified by the 1983 Eighth Constitutional Amendment (2). Over the following 35 years, a persistent struggle for abortion rights ensued in this predominantly Catholic nation (3). ROI gained notoriety for the “abortion trail” to the UK, with an estimated 5,000 people between 1990 and 1997 receiving abortions in the UK, prompting legal battles (4, 5). Two cases, *Attorney General v. X* in 1992 and the death of Savita Halappanavar in 2012, catalyzed changes in abortion access that are reflected in the ROI's legislation today (6).

In 2018, a landmark referendum led to the repeal of the Eighth Constitutional Amendment, permitting first-trimester abortions in ROI, reflecting a societal shift away from political and religious control over private life (7). A rapid rollout of physician training through the Irish College of General Practitioners (ICGP) facilitated centralized implementation (8). Currently, patients are able to receive free abortion care following a mandatory three-day waiting period and physician certification confirming gestational age. Beyond 12 weeks, abortion remains permissible with no gestational limit when there is a risk to life, serious harm to health, or when the fetus is unlikely to survive beyond 28 days after birth (9).

By comparison, the UK has had long established policies of abortion care under the 1967 Abortion Act, requiring two-physician approval under one of five legal grounds (10). Patients most commonly qualify under Ground C, preventing significant physical or mental harm to the pregnant person, up to 24 weeks’ gestation (11). Exceptions without gestational limits exist for cases involving grave risk to life, permanent injury to health, or severe fetal abnormalities (12).

Globally, nearly 60 countries have adopted legal reforms and offered greater protections on abortion care since 1994. The United States remains as one of four outlier countries that have regressed abortion regulations (1). In June 2022, the U.S. Supreme Court ruling in *Dobbs v. Jackson Women's Health Organization* overturned *Roe v. Wade* (1973). This decision removed the constitutional right to abortion in the United States, meaning that each state was to determine whether abortion is legal, and under what circumstances. In the aftermath, abortion access contrasted sharply. States like Texas (TX) reinforced the restrictions from the 2021 Texas Senate Bill 8 (“heartbeat bill”) and passed the 2022 Human Life Protection Act, enacting near-total bans on abortion. While patients may legally travel out of state for care, physicians who provide abortions within Texas face severe criminal penalties, including potential life imprisonment.

Meanwhile, states like California (CA) reinforced legal protections, passed shield laws, and expanded funding mechanisms to accommodate rising demand from out of state (13, 14). Abortion has been legal in CA since *People v. Belous (1969)* and is protected under the Reproductive Privacy Act, which allows abortion up to the point of fetal viability, with no gestational limit when a patient's life or health is at risk, or in cases of severe fetal anomaly (15). The 2023 Senate Constitutional Amendment 10 further enshrined abortion as a fundamental right within the California Constitution (16).

**Figure 1:**
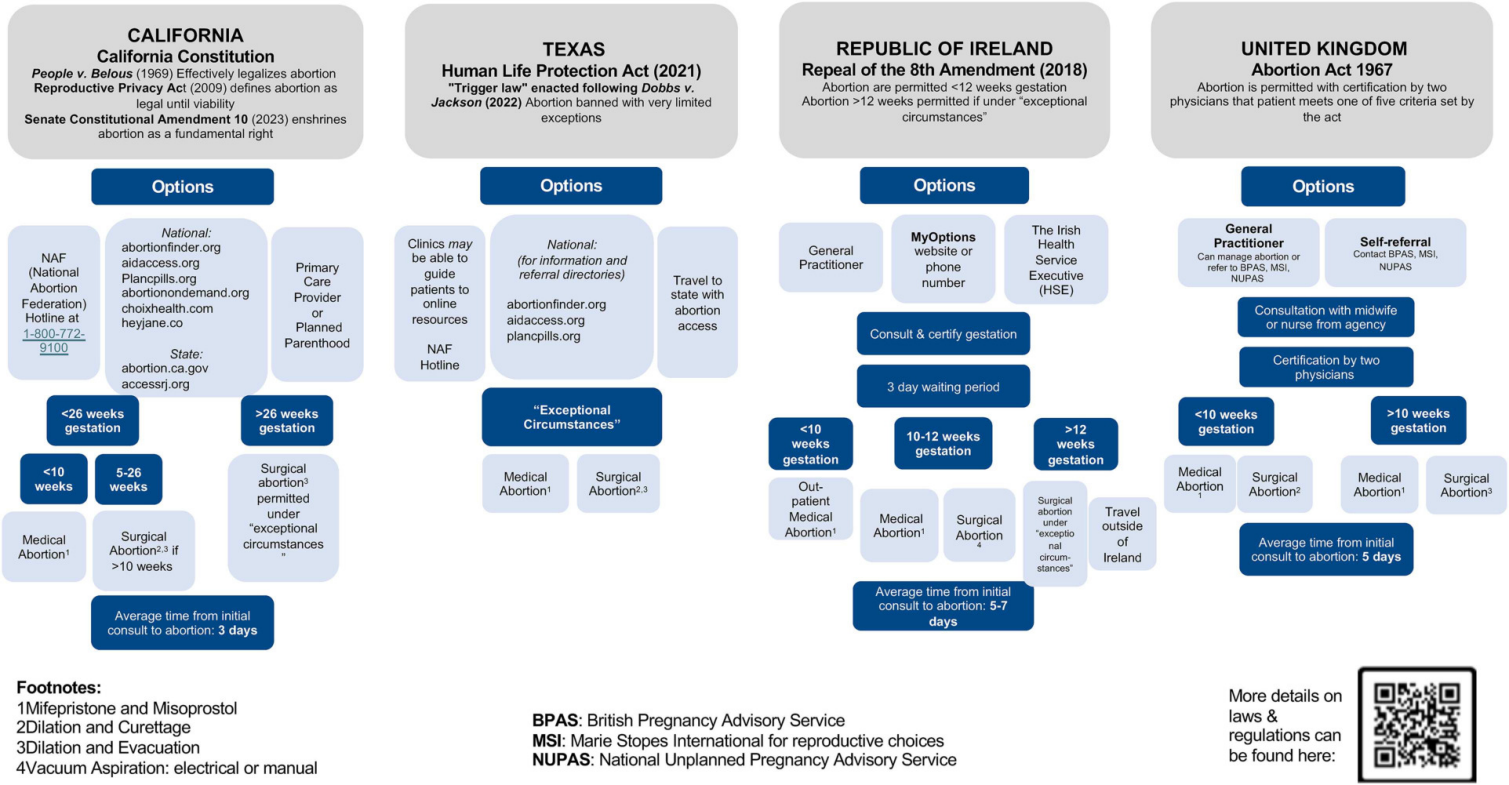
Overview of current pathways to abortion based on geographic location. Abortion is completely illegal in TX, whereas there are more options for obtaining an abortion in CA, ROI, and the UK (45, 46).

Logistics of obtaining care in each location, including sources of information, steps to take, and wait times are significantly altered in the context of legislative changes, healthcare infrastructure, and culture. Thousands of patients in more restrictive areas have crossed state lines for care (17), while others have turned to self-managed medication abortion often facilitated through telehealth (18, 19). Taking guidance from the historical relationship between ROI and the UK, who have navigated a shift from restrictive to more accessible abortion, we can anticipate and navigate ongoing changes in states like TX and CA (2). Figure 1 summarizes the current steps of obtaining care across each location of interest.

When interpreting physician perspectives in this study, it is important to account for differences in abortion care by gestational age. In the U.S., 93% of abortions in 2020 were performed in the first trimester (≤13 weeks), with either medication abortion up to 10 weeks, using mifepristone followed by misoprostol, or procedural abortion up to 13 weeks (20). First trimester abortions are considered very safe and effective, with most people recovering quickly, with a low risk of complications (21). Second trimester abortions (13–24 weeks) represent about 6% of cases, with options including dilation and evacuation, or less commonly, medical abortion using higher doses of mifepristone and misoprostol (20, 21). While second trimester abortions are generally safe, they carry a higher risk of complications requiring more specialized training and facility resources (21). Third trimester abortions at 27 weeks and beyond are rare, comprising less than 1% of all abortions (20). While shifting regulations have affected access to abortions across gestational ages, procedural abortions face greater barriers due to patient and physician-facing financial, social, logistical, and legal barriers (22). Although most abortions are first trimester, many of the physicians in this study speak to 2nd trimester procedural abortions in reflection of training, systemic barriers, and complex patient cases.

Drawing on these contextual realities, this study compares physician perspectives across CA and TX, alongside ROI and the UK, to examine how legislation shapes clinical practice and patient access across restrictive vs. protective environments.

## Methods

This qualitative interview study was conducted in accordance with the Consolidated Criteria for Reporting Qualitative Research (COREQ). The study received an exemption from the Institutional Review Board at the University of California, Davis. Interviews were conducted by four medical trainee researchers. To minimize bias, researchers engaged in regular reflexive discussions to examine how their own perspectives could shape data collection and interpretation.

A comparative case study design was utilized to examine physician experiences across four regions with varying regulations. Within the U.S., the focus was on CA and TX rather than attempting to cover the entire country as abortion policy varies widely at the state level. CA and TX were selected as representative states due to their size as large states with contrasting legal landscapes, with the former maintaining strong protections for abortion, and the latter enacting some of the most restrictive policies following the Dobbs decision. Internationally, ROI and the UK were included as ROI provided insight into a health system transitioning from restriction to liberalization following the 2018 repeal of the Eighth Amendment, and the UK offers long-standing abortion policies and served as the primary destination for Irish patients seeking care. These four locations serve to demonstrate meaningful contrasts in how legislation affects care.

A total of 19 abortion-trained physicians were recruited using purposive and snowball sampling, which was particularly useful given the limited number of physicians who publicly identify as providers of abortion care. Participants were drawn from CA (*n* = 6), TX (*n* = 4), the UK (*n* = 4), and the ROI (*n* = 5) (Table 1). Inclusion criteria included active or recent clinical experience in abortion care and English language fluency. We limited our study to physicians (MD, DO, MBBS, or equivalent) to focus our discussion on this group's training and scope of practice. Recruitment continued until thematic saturation was reached, meaning no new themes emerged in the final interviews.

**Table 1:** Characteristics of physicians interviewed.

Region	Specialty	Practice setting	Years in practice
California	Family medicine	Academic quaternary care hospital	5+
	Obstetrics & gynecology	Academic quaternary care hospital	10+
	Obstetrics & gynecology	Academic quaternary care hospital	10+
	Obstetrics & gynecology	Academic quaternary care hospital	15+
	Obstetrics & gynecology	Academic quaternary care hospital	20+
	Obstetrics & gynecology	Academic quaternary care hospital	25+
Texas	Obstetrics & gynecology	Academic quaternary care hospital	5+
	Obstetrics & gynecology	Academic quaternary care hospital	5+
	Obstetrics & gynecology	Public primary & preventive care	15+
	Obstetrics & gynecology	Public primary & preventive care	20+
United Kingdom	Obstetrics & gynecology	Academic quaternary care hospital	15+
	Obstetrics & gynecology	Academic quaternary care hospital	20+
	Obstetrics & gynecology	Academic quaternary care hospital	30+
	Obstetrics & gynecology	Academic quaternary care hospital	15+
Republic of Ireland	General practice	Rural community clinic	10+
	General practice	Metropolitan community clinic	15+
	General practice	Rural community clinic	20+
	General practice	Metropolitan community clinic	25+
	General practice	Metropolitan community clinic	35+

Each participant completed a one-hour, semi-structured interview conducted over Zoom or in person between August 2022 and November 2023. Verbal informed consent was obtained, including consent to audio-record the interview. An interview guide (Appendix 1) comprising ten open-ended questions was developed based on a literature review to ensure the topics of training, legislation, barriers to care, and practice environment were addressed in each interview. The guide was tailored to each region's legal landscape [e.g., *Dobbs v. Jackson Women's Health Organization* (2022) in the U.S., Eighth Amendment in ROI] and provided to participants prior to the interview to allow time for reflection.

All interviews were audio recorded and transcribed using Otter.ai transcription software. Transcripts were anonymized to preserve confidentiality and participants were assigned region-based codes (e.g., TX1, ROI2) for reference in reporting.

Thematic analysis was conducted following Braun and Clarke's six-step approach. Two researchers independently conducted open coding of the transcripts using an inductive approach. A shared codebook was developed collaboratively and refined through discussion. Discrepancies in coding were resolved through consensus. Coded data were grouped into broader categories and synthesized into key themes, subthemes, example codes, and illustrative quotes. Illustrative quotes were selected to represent the breadth and depth of identified themes when they captured the essence of a theme, reflected variation across locations, or provided particularly vivid or compelling language. The included interview quotes in this report are verbatim, with minimal edits for clarity, grammar, or length using standard conventions (e.g., brackets to insert text to clarify meaning, ellipses for omitted text).

## Results

Tables 2, 3 display themes, subthemes, codes, and representative quotations to evidence each theme gathered from interviews. The quotations are further contextualized in this section.

**Table 2:** Themes and sub-themes.

Themes	Sub-themes
Exposure to abortion care during general training	Sought external experiences
	Medical school exposure
	Residency exposure
	No exposure at all
Experience with dedicated abortion care training	Training after becoming an attending
	Full-scope abortion/family planning training during residency
	Build your own path in residency/optional training
Influence of laws and regulations on clinical practice and patient access	Legal restrictions on care provision
	Displaced patient load
	Gag policies and liability
	Adaptive workarounds
	Administrative burden
	Historic changes in policies
	Innovative reform
Individual level barriers to patient access	Lack of easy physical access to care
	Financial burden of abortion
	Emotional distress and patients experience
	Patient identities and socioeconomic status
Systems level barriers to patient access	Facility associated barriers
	Professional identities and moral sets
	Healthcare infrastructure
	Stigma within systems of care
Physicians’ roles in advocacy and legislative guidance	Advocacy beyond obstetrics and gynecology
	Responsibility for public education
	Empowering patient voices
	Building early advocacy through medical education
Physician-facing stigma	Maintaining patient-centered care despite personal beliefs
	Selective disclosure of professional identity
	Experience with protesters and dissent
	Isolation and moral fatigue experienced by physicians
Common misconceptions	Safety profile of abortion procedures
	Patient motivations for seeking abortion care
	Training, qualifications, and quality of abortion care physicians
	Rhetoric surrounding abortion

**Table 3:** Sub-themes, codes, & supporting quotations.

Sub-themes	Codes	Supporting quotations
Sought external experiences	Lack of curriculum; fellowship-based training	[TX] “I had to seek out my own training in residency. We did not have a formal curriculum regarding family planning. So I ended up doing an away rotation at [an outside hospital] for I think it was about a month or so in my third year of residency..I got the bulk of my abortion training [in fellowship].”
Medical school exposure	Student advocacy; early procedural exposure; externship	[UK] “When I was in medical school, I got involved with Medical Students for Choice..I did a reproductive health externship…at a Planned Parenthood in California, and you would never be able to do this now, but I did my first abortion as a medical student.”
Residency exposure	Integrated curriculum	[CA] “[My program] was one of the few programs in the country that had family planning as a standard rotation. I went to the residency to become a high risk obstetrician. And in my third year of residency, I did my family planning rotation and I found my home because…I always wanted to, within medicine, find people who really needed care and provide that care.”
No exposure at all	Abortion illegal; blank canvas; lack of hands-on training	[ROI] “It was illegal. It was absolutely not part of our training. And until the legislation was passed in 2018, we had no idea so we were a blank canvas in a way. I'm sure it has changed immensely for younger doctors doing their training now, especially for doctors in GP training, but for those of us who are established GPs already in the community, it wasn't there. There was no hands-on training.”
Training after becoming an attending	Postgraduate workshop; peer support; confidence-building	[ROI] “The ICGP provided the training, it was a one day course from ten [AM] to four [PM] and it was very straightforward…It just gave us the confidence to do it. Because before you do a service like this, you always think it's going to be more complicated than it actually is..The training was very easy, but actually it's the follow-up support that we get from our WhatsApp group which is invaluable for me and for most people, I think.”
Full-scope abortion/family planning training during residency	Comprehensive care; ethics; well-rounded education	[TX] “Medication abortion, procedural abortion, much more well rounded pregnancy options, counseling, ethics of abortion care, ethics of just healthcare in general…I am so thankful that I had that training because I think that really has allowed me to be a much better physician today.”
Build your own path in residency/optional training	Self-directed modules; second trimester access; inter-hospital	[UK] “In your last few years, you do your core training and some specialized modules in whatever area you plan to practice in. So I took an advanced training module in abortion care…which only provided surgical terminations up to 15 weeks at that point. So I had to go elsewhere then to get some training in second trimester procedures. So I went to a couple of other units in London to do that. But [training was] very readily provided by the few people that do provide it, so once you know where to look, the training was fairly easily accessed.”
Legal restrictions on care provision	State bans; loss of clinical autonomy; inability to provide care for lethal fetal anomalies	[CA]“The Dobbs Decision has affected everyone..This legal decision has fundamentally changed the relationship between medical providers and their patients and to what degree it is dictated by legislation.”
Displaced patient load	Spillover effects; demand of care; cross-state travel	[CA] “I expect that over the next several months, we will see more at this institution, and I think that's more of an overflow situation. For example, patients in Texas go to Oklahoma, and then patients in Oklahoma that had overflow go to a different state because all their appointments were already used for these patients who had left Texas. And so we started seeing the sort of overflow and then over into New Mexico, then from New Mexico to Southern California.”
Gag policies and liability	Institutional censorship; inability to counsel; threat of prosecution	[TX] “Our hospital has actually advised all of the faculty to be very vague in regards to talking about abortion care..I do not think that any state legislation should be in the way of me speaking to my patient about evidence-based and appropriate care.”
Adaptive workarounds	Patient navigation, peripheral resources, out of state abortion	[TX] “Essentially, [we now] provide peripheral resources that are legitimate so that patients can seek out-of-state legal abortion.”
Administrative burden	Two-doctor approval; functional permissiveness	[UK] “[It's] one of the only areas in UK medicine where doing something that would be essentially routine health care would be illegal unless you do the paperwork properly. So it's quite a big deal that there's this burden that you have to do it. However, I think the thing to understand about UK culture is…the superficial appearance is not necessarily the reality on the ground…the reality is a fairly permissive structure.”
Historic changes in policies	Sociocultural transformation; normalization of care	[ROI] “Prior to the Eighth Amendment [referendum], we voted on the right to information and the right to travel, which now just seems absurd. I believe it was illegal to talk about abortion to women as a GP.”
Innovative reform	Telemedicine; expanded access	[UK] “The woman would take the mifepristone and misoprostol at home without ever having to come in.”
Lack of easy physical access to care	Transportation issues; rural burden; geographic distance	[CA] “There was a patient who was initially 14-weeks along coming from somewhere very far. Her car broke down and she had to be rescheduled to the following week. The following week, her car was still in the shop. The friend that was supposed to give her a ride refused after learning that she was going to get an abortion. The following day, she had to take three buses over 5 h to get to clinic, and by then, she was in her second trimester. We usually don't admit people to the hospital after dilators, but there was no way she was going to get back. She had obviously been through hell and high water to get here. So that's when the social determinants of health were really clear.”
Financial burden of abortion	Affordability; missed work; childcare cost; cost of travel	[TX] “They do have to travel for care, which is incredibly costly..affording a procedure, affording the time off from work, affording the childcare, affording how you get there, all of those things.”
Emotional distress and patient experience	Shame; isolation; travel trauma	[IR] “Bottom line is, women have been getting abortions in this country for as long as they have been in any other country. But they've had to travel. There's a certain cruelty about that and there's a loneliness and there's a shame.”
Patient identities and socioeconomic status	Cultural norms, linguistic barriers, educational inequities	[UK]“There's a huge number of women that socially and culturally [can't] access care and have little choice.”
Facility associated barriers	Appointment availability, scheduling delays, wait times	[CA]“We only do procedures Tuesdays and Fridays and then need to do a pre-op appointment… sometimes we are scheduling out three weeks.”
Professional identities and moral sets	Physician conscientious objection; lack of referral follow-up	[ROI]“Whether the GP is consciously or unconsciously against abortion, they haven't referred them to the right place..instead of a simple procedure in a GP practice, they have to go to the hospital.”
Healthcare infrastructure	Undercapacity; private sector limitation; systems bottlenecks	[UK] “Where barriers at the NHS and independent sector interact is around people who have medical complexities that need to move from the independent sector into hospital settings. [People especially in the] second trimester probably face the most barriers of all, so if you're very late in the second trimester, and you have a medical complexity, which means you can't be treated at BPAS, you may very well get turned away.”
Stigma within systems of care	Implicit bias; judgment	[UK] “The last thing they need is me judging them. And the number of women that cry and go “Oh, you guys are so nice. I didn't expect to be treated with so much respect and kindness…”The expectation is that they will be judged that much just when they're seeking health care.”
Advocacy beyond obstetrics and gynecology	Cross-specialty advocacy; unified voice in medicine	[TX]“We are past the point now of staying silent. This affects every aspect of medicine, not just OB-GYN, family medicine, or emergency medicine. Every single field of medicine is negatively impacted by these laws.”
Responsibility for public education	Legislative awareness; clinically informed policy	[CA]“We have a responsibility to object to laws and regulations that compromise patient safety. That can mean public advocacy, but it can also mean educating colleagues and policymakers. It shouldn't just be OB-GYN or family medicine, every specialty is affected. Emergency rooms will see the complications of self-managed abortions, and labor floors will see more unintended pregnancies.”
Empowering patient voices	Patient-centered advocacy; listening to lived experiences	[UK]“Those of us engaged in abortion care can [voice patient experiences] because often our patients don't feel able to do it for themselves. Doctors can play a powerful role in giving voice to the experience of patients and describing what they need.”
Building early advocacy through medical education	LEGISLATIVE education; professional responsibility	[UK]“A colleague is working to standardize abortion care training in all medical schools. Getting students to think about abortion care early helps them become conscientiously committed for the rest of their careers. Many have gone on to join Doctors for Choice UK and other advocacy groups.”
Maintaining patient-centered care despite personal beliefs	unbiased support; autonomy; ethical stance	[CA] “I don't understand all of the nuances of people's life circumstances and so honestly, I don't really need to know why somebody is terminating the pregnancy to help them get the care that they need.”
Selective disclosure of professional identity	Selective disclosure; physician safety	[TX] “There's a pretty wide variety of people's comfort and my own comfort is that I don't disclose my job or my fellowship training in general to people unless I know them and I kind of know their viewpoint. In an academic setting at my hospital, I'm happy to do so because I'm in my role and it's part of my training and who I am as a doctor, but not in my personal life.”
Experience with protesters and dissent	Protestors; public dissent	[CA] “Every day we go to Planned Parenthood, we face the protesters who call us murderers and psychopaths and compare us to serial killers…they were telling me I was the same as [a] mass shooter. It doesn't necessarily impact my practice, but it certainly affects you from a professional and personal perspective. We're trained not to interact with the protesters so you just kind of keep your head down, your glasses on, your mask on, and your earphones in and try to walk by.”
Isolation and moral fatigue experienced by physicians	Moral fatigue; professional isolation	[CA] “Sometimes we feel like we're the only ones fighting for our patients to get care, and like hitting a lot of barriers.”
Safety profile of abortion procedures	Safety misconceptions; politicization of medicine	[CA] “Because abortion is so politicized, everybody thinks it's really dangerous. It is safer than a colonoscopy, safer than some dental procedures from a mortality perspective, and safer than if somebody were to continue their pregnancy and have a vaginal delivery or a C-section at term. People get sucked into all the political mumbo jumbo and don't realize that it is one of the simplest and safest procedures in medicine.”
Patient motivations for seeking abortion care	Respect for decision making	[ROI] “A lot of people may believe that people who seek abortions do not see it as a big thing. Whereas in my experience, they do…People in Ireland are very careful about their contraception and they don't see abortion as a safety net.”
Training, qualifications, and quality of abortion care physicians	Misconceptions about physician competence	[Texas] “The idea that abortion providers are the bottom of the barrel when it comes to doctors, that we provide this care because we're not good enough to do anything else, [we are] people who didn't do well in medical school or do well in their residency, are bad surgeons, or can't get any other jobs, couldn't be farther from the truth. People are incredibly accomplished and compassionate.”
Rhetoric surrounding abortion	Normalizing abortion language	[CA] “Every pregnancy terminates, a C-section is a pregnancy termination. A delivery is a pregnancy termination. So call it what it is, it's an abortion, and it's okay to use that word”.

### Abortion care training

Participants described variable exposure to abortion care throughout their training, reflecting how legislation, geographical, and institutional culture shape access to training. Individual initiative during medical school allowed for great exposure as described by a UK physician:

“When I was in medical school, I got involved with Medical Students for Choice..I did a reproductive health externship…at a Planned Parenthood in California, and you would never be able to do this now, but I did my first abortion as a medical student.”

However, this early procedural exposure was more of an exception, not the norm in early training. Participants in the U.S. and UK emphasized that they often sought out extracurricular engagement before obtaining more formal training through residency. A California-trained physician reflected:

“There were no opportunities for a medical student like myself to be a part of abortion care, but I was a part of Medical Students for Choice…I went to Planned Parenthood for a couple of days to observe the clinic there…In OB-GYN residency, we were a Ryan Program. Luckily, I specifically wanted an OB-GYN program that incorporated abortion services into the curriculum.”

Across all geographic locations, abortion care was not a standardized component of residency training either. Some programs offered structured opportunities embedded in residency while others required trainees to independently seek out experiences. A CA physician noted how program structure shaped professional identity:

“[My program] was one of the few programs in the country that had family planning as a standard rotation. I went to the residency to become a high risk obstetrician. And in my third year of residency, I did my family planning rotation and I found my home because…I always wanted to, within medicine, find people who really needed care and provide that care.”

In contrast, many trainees had to build their own path in residency to gain exposure and competency. Two TX participants explained their experiences of electing to obtain training in abortion care:

“We had an opt-in time where we could go to Planned Parenthood and participate in care. The time for you to go was there, but it wasn't necessarily an integrated part of the curriculum.”

“I had to seek out my own training in residency. We did not have a formal curriculum regarding family planning. So I ended up doing an away rotation at [an outside hospital] for I think it was about a month or so in my third year of residency..I got the bulk of my abortion training [in fellowship].”

Similarly, a UK physician described pursuing advanced modules across institutions to fill curricular gaps. One participant explained pursuing this particular interest as such:

“..I took an advanced training module in abortion care…which only provided surgical terminations up to 15 weeks at that point. So I had to go elsewhere then to get some training in second trimester procedures. So I went to a couple of other units in London to do that. But [training was] very readily provided by the few people that do provide it, so once you know where to look, the training was fairly easily accessed.”

Even among programs with integrated rotations, exposure beyond first trimester care was often self-directed. Those with access to full-scope training described it as transformative. One Texan reflected:

“Medication abortion, procedural abortion, much more well rounded pregnancy options, counseling, ethics of abortion care, ethics of just healthcare in general…I am so thankful that I had that training because I think that really has allowed me to be a much better physician today.”

In contrast, in legally restrictive environments such as pre-2018 ROI, physicians longer in practice described how abortion training was absent when they initially trained.

“It was illegal. It was absolutely not part of our training. And until the legislation was passed in 2018, we had no idea so we were a blank canvas in a way. I'm sure it has changed immensely for younger doctors doing their training now, especially for doctors in GP training, but for those of us who are established GPs already in the community, it wasn’t there. There was no hands-on training.”

Following the repeal of the Eighth Amendment, the ROI rapidly implemented a national training program through the Irish College of General Practitioners (ICGP). An Irish GP described the program's benefits, including the confidence and ongoing support it provided:

“The ICGP provided the training, it was a one day course from ten [AM] to four [PM] and it was very straightforward…It just gave us the confidence to do it. Because before you do a service like this, you always think it’s going to be more complicated than it actually is..The training was very easy, but actually it’s the follow-up support that we get from our WhatsApp group which is invaluable for me and for most people, I think.”

In the context of ongoing changes in abortion policies across the U.S., physicians in both TX and CA expressed concern that the gaps in abortion training will create future risks. One physician warned:

“If things don't change..there’s going to be a huge gap in knowledge between people who were trained in some places vs. others. That’s going to be really scary when half your OB-GYNs are not familiar with abortion and abortion complications in 10 years.”

This loss of procedural skill carries real risks for patient safety. A TX physician explained:

“Dilation and curettage is a bread-and-butter procedure of gynecology, and the procedure is like a first-trimester abortion…The ability to empty a uterus of any size, quickly and efficiently, is an incredibly important skill set..because, at any stage, that pregnancy can very quickly become very dangerous for the pregnant person. [People] who have a potentially life threatening emergency who need to have their uterus empty, and the only thing we can do is offer them an induction or hysterectomy, is ridiculous. It really is a life saving skill.”

While participants described acquiring the technical skills for uterine evacuation for other indications, their comfort with counseling abortion care varied. Beyond procedural skills, the ability to adequately counsel patients and help them navigate their decisions will also be lost. A TX physician specifically noted:

“Some of the things that you don't learn about as well if you don't do abortion care specifically is how to have those conversations with patients regarding abortion or an unplanned or undesired pregnancy. If you're in a place where everyone assumes every pregnancy is desired, it’s hard to get experience talking to people about that in a comfortable and appropriate way.”

### Influence of laws and regulations

Beyond adequate knowledge and training, perhaps one of the greatest barriers involves the challenges of providing comprehensive reproductive care within the context of a region's legal framework. In CA, participants noted that while their ability to provide abortion care remained protected, they increasingly saw patients traveling from more restrictive states. One explained:

“The Dobbs Decision has affected everyone..This legal decision has fundamentally changed the relationship between medical providers and their patients and to what degree it is dictated by legislation.”

Participants in CA additionally described “spillover” effects, illustrating how state restrictions are felt nationally, displacing patient load rather than eliminating demand:

“I expect that over the next several months, we will see more at this institution, and I think that’s more of an overflow situation. For example, patients in Texas go to Oklahoma, and then patients in Oklahoma that had overflow go to a different state because all their appointments were already used for these patients who had left Texas. And so we started seeing the sort of overflow and then over into New Mexico, then from New Mexico to Southern California.”

In TX, the most significant barrier was the post-Dobbs Decision ban on abortion provision, including for life-threatening or lethal fetal conditions or for maternal health. One participant summarizes this:

“In the 14 years that I've been here, how you get to provide an abortion in the state of Texas has changed dramatically…The fact that the system now says that you cannot provide abortion at all is the biggest barrier.”

As a result, “gag rules” limit the information that can be disseminated to patients. Physicians described the effect of the restrictive laws on patient awareness and the ability of physicians to provide counseling:

“Our hospital has actually advised all of the faculty to be very vague in regards to talking about abortion care..I do not think that any state legislation should be in the way of me speaking to my patient about evidence-based and appropriate care.”

As a downstream effect, one physician described how their institution, previously performing 8,000–10,000 abortions annually, downsized dramatically:

“We've also had to sell our surgery center because we can't afford to maintain that [when we’re] not providing that care anymore.”

Despite these barriers, some TX physicians found ways to provide support through ancillary services like patient navigators, dating ultrasounds, and resources like gas cards, plane tickets, and childcare:

“Essentially, [we now] provide peripheral resources that are legitimate so that patients can seek out-of-state legal abortion.”

In contrast to recent legislative changes in the U.S., UK physicians described how the country's long-standing legal framework shapes abortion access. One explained that the requirement for two doctors’ approval adds administrative burden but rarely limits care:

“[It’s] one of the only areas in UK medicine where doing something that would be essentially routine health care would be illegal unless you do the paperwork properly. So it’s quite a big deal that there’s this burden that you have to do it. However, I think the thing to understand about UK culture is…the superficial appearance is not necessarily the reality on the ground…the reality is a fairly permissive structure.”

They noted that, despite the appearance of tight regulation, abortion under 24 weeks is effectively available under Ground C, which allows abortion if two doctors believe that continuing pregnancy poses a risk to the physical or mental health of a pregnant person. One physician explains how these terms are always true:

“It’s always risky to be pregnant at 32 weeks as it is 12 weeks. So by default, you end up [qualifying] and so as long as there are two people in the country who know who you are, doesn't [have to] know you personally, they just have to know that you are a woman who is less than 24 weeks who doesn't want to be pregnant…that ground becomes true.

Participants also described the impact of the provisional “Pill by Post” program where due to quarantine measures during the COVID-19 pandemic, patients were assessed via telehealth appointments and medications for medical abortions were sent to patients’ residence for at-home administration. A physician explained that under the provisional program:

“The woman would take the mifepristone and misoprostol at home without ever having to come in.”

The “Pills by Post” provisional program was slated to end; however, following public and professional support, the program became permanent. Another physician highlighted that prior to this program if…

“someone [was] severely agoraphobic and couldn't go into clinic [had] accessed pills online, and if caught, [they] could face serious consequences.”

In ROI, physicians described the shift in ability to provide, as well as attitudes towards abortion. One general practitioner reflected on when abortion was both legally and culturally restricted, with limited ability to even discuss it in clinical settings:

“Prior to the Eighth Amendment [referendum], we voted on the right to information and the right to travel, which now just seems absurd. I believe it was illegal to talk about abortion to women as a GP.”

Before legalization, many recounted the limitations of what they could provide for patients seeking an abortion.

“Before the legalization of abortion, all I could have done is give people a number for an English provider and said this is where you can go.”

While some physicians remained personally influenced by their Catholic upbringing after legalization, they recognized the importance of providing this care. Many framed their work as delivering a service legally affirmed by the Irish public:

“Like a lot of Irish Catholics, the thought that abortion is wrong is deep seated in you. But I have changed. Legally, it means you are just providing something that was legally voted in by people of Ireland.”

### Barriers to care beyond legislation

**Figure 2:**
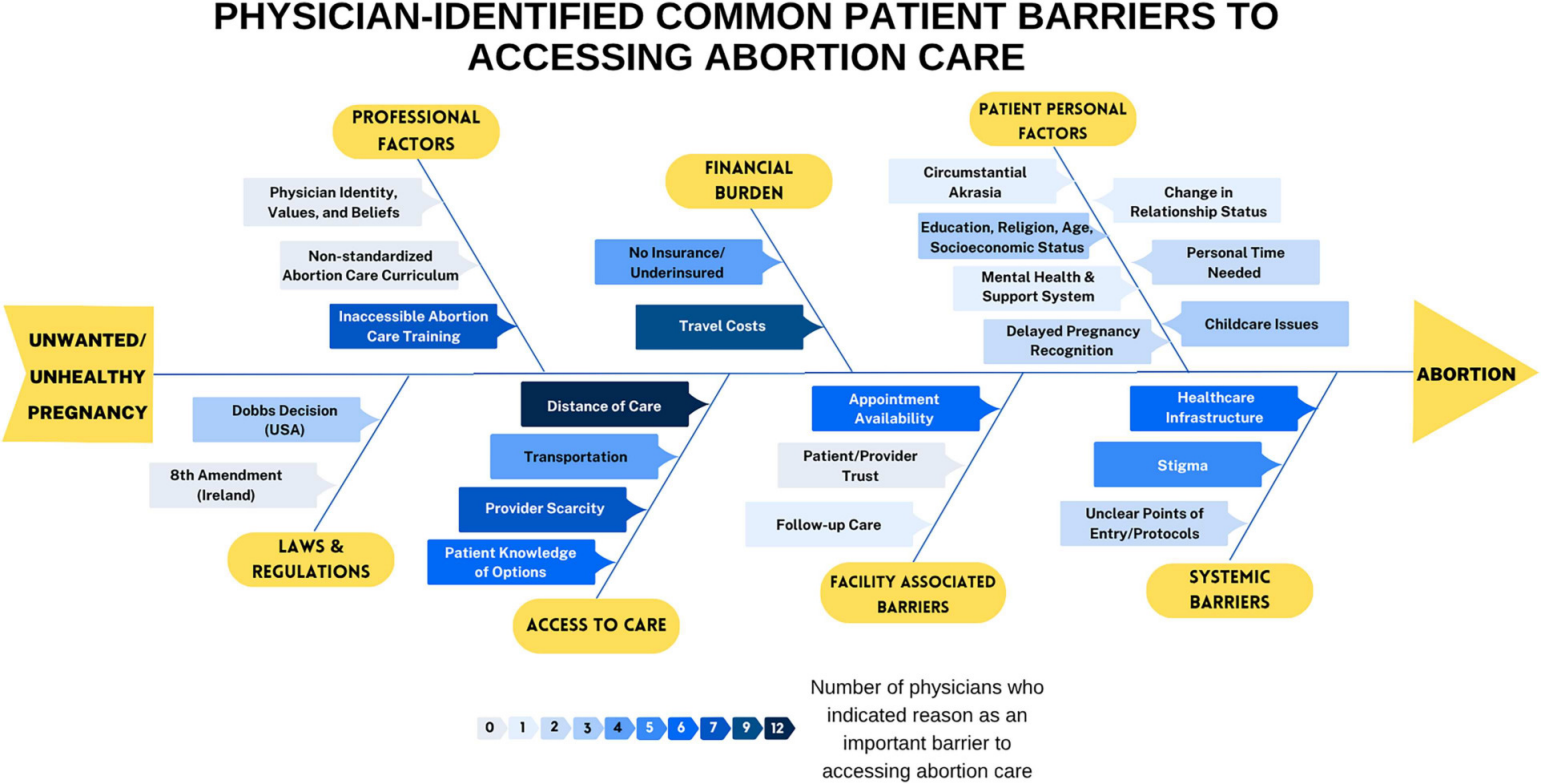
Fishbone diagram and heat map highlighting barriers to abortion care. The darker the blue color, the more often that barrier was mentioned during interviews.

Aside from policies dictating care provision, interviewees across all locations identified multiple intersecting barriers that patients face when seeking abortion care, ranging from personal barriers to structural and health system obstacles. Figure 2 summarizes these barriers across locations. Although these constraints vary across legal contexts, they collectively reveal the interplay of systemic inequities with personal factors preventing care even when it is legally protected.

Distance of care and transportation modality were major determinants of access, especially in rural locations and regions with few procedural sites. One Californian physician detailed a patient experience related to this:

“There was a patient who was initially 14-weeks along coming from somewhere very far. Her car broke down and she had to be rescheduled to the following week. The following week, her car was still in the shop. The friend that was supposed to give her a ride refused after learning that she was going to get an abortion. The following day, she had to take three buses over 5 h to get to the clinic, and by then, she was in her second trimester. We usually don't admit people to the hospital after dilators, but there was no way she was going to get back. She had obviously been through hell and high water to get here. So that’s when the social determinants of health were really clear.”

A similar geographic barrier was noted in the UK, where physicians named limited NHS infrastructure for late gestation procedures:

“There aren't enough National Health Service (NHS) hospitals that provide the full range of [abortion] services, [with only] five surgical centers in the country that can do the late gestation…and four of them are in London.”

Similar to distance, cost remained a pervasive barrier even when the abortion itself was legally available. A Texan physician emphasized how this financial strain compounds inequity in care:

“They do have to travel for care, which is incredibly costly..affording a procedure, affording the time off from work, affording childcare, affording how you get there, all of those things.”

Financial burden also arises in the form of insurance restrictions. A TX physician recounted a case that illustrated a disconnect between how reproductive care and general healthcare are classified by insurance policies:

“[She was] affected by a lethal fetal condition. And she chose to terminate the pregnancy and had to go to an abortion clinic outside our city because it’s not something that was covered by her insurance. And to me, while she ended up receiving excellent care at an excellent facility, [I saw] how separate her medical care for her pregnancy had become once she decided to have an abortion.”

In contrast, an Irish participant noted that while procedures are free of charge, travel remains a significant issue due to an urban concentration of physicians, similar to in the UK. This physician highlights:

“Financial burden [of the procedure itself] isn't really an issue as it’s all completely free.”

Californian participants pointed out facility-related barriers, including scheduling challenges, that is also echoed by providers across regions. One provides an example:

“We only do procedures Tuesdays and Fridays and then need to do a pre-op appointment…sometimes we are scheduling out three weeks.”

Beyond tangible barriers, physicians spoke to the emotional toll and isolation experienced by patients forced to seek care abroad or in secret. An Irish participant described:

“Bottom line is, women have been getting abortions in this country for as long as they have been in any other country. But they've had to travel. There’s a certain cruelty about that and there’s a loneliness and there’s a shame.

Physicians across contexts shared stories of patients expecting judgment in treatment, adding a layer of complexity to seeking care. A UK physician noted:

“The last thing they need is me judging them. And the number of women that cry and go ‘Oh, you guys are so nice. I didn't expect to be treated with so much respect and kindness…’ The expectation is that they will be judged that much just when they're seeking health care.”

Across regions, participants noted how personal factors and identities such as education, religion, and socioeconomic status compounded inequities in care. One UK participant explained:

“There’s a huge number of women that socially and culturally [can’t] access care and have little choice.”

Another described the persistence of linguistic barriers:

“For all the patients I see that we have interpreting services for..there must be far more that don't access any care at all.”

In ROI, professional identities and moral sets further hinder care, with many GPs not providing follow-up services. A participant described:

“Whether the GP is consciously or unconsciously against abortion, they haven't referred them to the right place..instead of a simple procedure in a GP practice, they have to go to the hospital.”

Similar to ROI, UK physicians also highlighted latent conscientious objection, where NHS physicians often avoid involvement in abortions without formally objecting, creating hidden barriers. Additionally, the NHS is rarely commissioned to provide abortions due to lack of profitability. Most abortions are performed in the independent sector, but when private hospitals face challenges in handling medical complexities, issues ultimately arise when patients need to transition to NHS care. One physician described this common challenge:

“Where barriers at the NHS and independent sector interact is around people who have medical complexities that need to move from the independent sector into hospital settings. [People especially in the] second trimester probably face the most barriers of all, so if you're very late in the second trimester, and you have a medical complexity, which means you can't be treated at BPAS, you may very well get turned away.”

Thus, bottlenecks often arise from navigating between public and private healthcare.

### Role of physicians in guiding abortion care legislation

Physician interviewees across regions voiced concerns regarding the politicization of clinical care. One participant drew a stark analogy:

“You don't want a legislator in the exam room when you're deciding your cancer treatment, you don't want a legislator in the room when you're at your well-child visit. You don't need any legislator in the room for anything. They're not medical providers.”

Physicians emphasized that the responsibility for advocacy should extend beyond OB-GYN to physicians of all specialties as limiting access to pregnancy termination services negatively impacts all fields of medicine. As one TX physician explained,

“We are past the point now of staying silent. This affects every aspect of medicine, not just OB-GYN, family medicine, or emergency medicine. Every single field of medicine is negatively impacted by these laws.”

Across countries, physicians described the importance of advocacy and education to ensure that lawmakers and the public understand clinical realities. A physician from ROI reflected,

“People should be listening to patients, to their partners, to people that love them. Lawmakers should be listening to physicians and what we’re seeing day in and day out.”

A UK physician also echoed the sentiment that doctors can play a powerful role in amplifying patient voices:

“Those of us engaged in abortion care can [voice patient experiences] because often our patients don't feel able to do it for themselves. Doctors can play a powerful role in giving voice to the experience of patients and describing what they need.”

Participants described several ways physicians can engage, from advocacy to legislative education. A Californian physician explained:

“We have a responsibility to object to laws and regulations that compromise patient safety. That can mean public advocacy, but it can also mean educating colleagues and policymakers. It shouldn’t just be OB-GYN or family medicine, every specialty is affected. Emergency rooms will see the complications of self-managed abortions, and labor floors will see more unintended pregnancies.”

Similarly, a UK physician described how advocacy can begin through education and mentorship:

“A colleague is working to standardize abortion care training in all medical schools. Getting students to think about abortion care early helps them become conscientiously committed for the rest of their careers. Many have gone on to join Doctors for Choice UK and other advocacy groups.”

### Physician-facing stigma and common misconceptions of abortion care

Across regions, physicians shared stories demonstrating various degrees of stigma surrounding abortion care, both in how they are perceived as physicians and how abortion itself is discussed within medicine. Participants emphasized grounding their practice in patient autonomy, even when personal belief systems or societal attitudes differ. A CA physician reflected:

“I don't understand all of the nuances of people’s life circumstances and so honestly, I don't really need to know why somebody is terminating the pregnancy to help them get the care that they need.”

Participants discussed how stigma affects their professional and personal lives, shaping the circumstances around when they disclose their professional role. As one TX physician shared:

“There’s a pretty wide variety of people’s comfort and my own comfort is that I don't disclose my job or my fellowship training in general to people unless I know them and I kind of know their viewpoint. In an academic setting at my hospital, I'm happy to do so because I'm in my role and it’s part of my training and who I am as a doctor, but not in my personal life.”

Other physicians described the extent of hostility from protestors outside clinics and the response they were trained to take. One CA physician noted:

“Every day we go to Planned Parenthood, we face the protesters who call us murderers and psychopaths and compare us to serial killers…they were telling me I was the same as [a] mass shooter. It doesn't necessarily impact my practice, but it certainly affects you from a professional and personal perspective. We're trained not to interact with the protesters so you just kind of keep your head down, your glasses on, your mask on, and your earphones in and try to walk by.”

The effects of public dissent take an emotional toll over time, contributing to moral fatigue, and serving as a reminder to compounded barriers to care:

“Sometimes we feel like we're the only ones fighting for our patients to get care, and like hitting a lot of barriers.”

Given the ongoing discourse surrounding changing legislation in the United States, U.S. based physicians shared many stories of public dissent. Additionally, a UK physician gave an account comparing U.S. protest experiences with those in London:

“Politics [in the UK] are not infused with religion in the same way [as in the U.S.]. There is greater respect for protecting individuals’ rights to make decisions for themselves. The anti-abortion movement exists here…but they don't get as much attention. I went to one of our clinics in London after they passed the buffer zone, and there was no money around, no protesters around, there was one woman across the street with the rosary. [The buffer zone] was three to four blocks down the way. It was huge. Those things have been more successful here than in the U.S.

In addition to stigma, physicians across all regions highlighted widespread misconceptions about abortion care. Many emphasized that abortion is a safe and routine medical procedure, contrasting public rhetoric with clinical reality.

“Because abortion is so politicized, everybody thinks it’s really dangerous. It is safer than a colonoscopy, safer than some dental procedures from a mortality perspective, and safer than if somebody were to continue their pregnancy and have a vaginal delivery or a C-section at term. People get sucked into all the political mumbo jumbo and don't realize that it is one of the simplest and safest procedures in medicine.”

Participants also challenged assumptions about the motivations of people seeking abortions. An Irish physician explained that most patients approach the decision with thoughtfulness and care:

“A lot of people may believe that people who seek abortions do not see it as a big thing. Whereas in my experience, they do…People in Ireland are very careful about their contraception and they don't see abortion as a safety net.”

Aside from misconceptions about the procedure or patients, a TX physician spoke to misconceptions about the physicians themselves:

“The idea that abortion providers are the bottom of the barrel when it comes to doctors, that we provide this care because we're not good enough to do anything else, [we are] people who didn't do well in medical school or do well in their residency, are bad surgeons, or can't get any other jobs, couldn't be farther from the truth. People are incredibly accomplished and compassionate.”

Finally, participants described how reclaiming and normalizing the language around abortion can help reframe how the public and the medical community perceive the procedure. A California physician clarifies:

“Every pregnancy terminates. A C-section is a pregnancy termination, a delivery is a pregnancy termination, so call it what it is. It’s an abortion, and it’s okay to use that word.”

## Discussion

This study explores how legislation, training structure, and social context all intersect to shape abortion care across four regions: California (CA), Texas (TX), the United Kingdom (UK), and the Republic of Ireland (ROI). Perspectives across several interconnected domains including training, laws and regulations, barriers to care, physicians’ roles in advocacy, and stigma and misconceptions were elicited from participants to demonstrate clinical realities of care across locations. Although physician commitment to patient-centered care remained consistent across settings, the structural and legal contexts in which they operate shaped every stage of care delivery.

### Variations in training access and future implications

Participants described variable training in abortion care across all stages of training, reflecting how legality, social context, and culture influence whether abortion is seen as standard medicine vs. optional knowledge. Physicians practicing in legally restrictive environments, such as TX and pre-2018 ROI, faced significant barriers to abortion training. Many physicians described “building their own path,” seeking out external rotations, fellowships, or informal support networks to gain competency. In contrast, physicians trained in CA, the UK, and post-reform ROI described more integrated, accessible, and systems-supported pathways. One such example is through the U.S. OB-GYN Ryan Residency Training Programs, which are dedicated to providing full-scope training in reproductive care (23) and the Irish College of General Practitioners’ (ICGP) national training (8, 24).

These differences in training access were mirrored by patient experiences: in restrictive settings, patients frequently faced greater travel, financial, and emotional strain, while physicians described moral distress when unable to provide patient-centered, medically indicated care (25). Participants warned that the lack of training causes downstream effects of inadequate procedural skills and a lack of counseling confidence, compromising patient safety. This demonstrates that gaps in training and patient inequities in knowledge and access are structurally linked, emphasizing the importance of incorporating abortion education through all steps of medical training (26).

### Legislation as a structural determinant of care

The physician experiences shared through this study illustrate how legal frameworks actively shape how care is discussed and delivered. In restrictive environments like TX, the clinical landscape has been transformed to one of fear, uncertainty, fragmentation, and moral injury (27, 28). Physicians commented on the drastic shifts in their ability to provide assistance to patients, the closure of facilities, and ultimately the erosion of trust in the patient-physician relationship. In the confines of law, physicians have shifted their roles to be more ancillary in nature, though the fear of repercussions for assisting individuals seeking abortion has made these roles vague and limit any significant help (29).

As a parallel, California offers a legal protection that does not automatically ensue equity in accessibility. While continued strong safeguards for abortion allow the state to serve in and out of state patients, this expansion adds new infrastructure pressure. Longer wait times, lack of provider availability, and systemic overload can arise when progressive states absorb the consequences of a post-Dobbs America (30).

In contrast to the fragmented U.S. response, the ROI offers both cautionary and instructive lessons on how legislation can reshape healthcare delivery. The centralized and coordinated policy response to the repeal of the Eighth Amendment helped destigmatize abortion care and normalize it within general practice. This multi-pronged, community-oriented model of care included national referral guidelines, rapid rollout of physician training through the Irish College of General Practitioners, and the development of peer support systems to facilitate implementation (9). ROI's reform demonstrates how legal change can allow for professional and cultural transformation when paired with a unified mechanism for education and healthcare infrastructure.

In comparison, the UK's longstanding framework for abortion care offers an example of stability in policy. Although it served as a critical safety net for Irish individuals seeking abortion before the legislative changes in their own country, in some respects, current legislation in ROI appears to offer more straightforward access than in the UK, where there is an administrative burden. However, despite the two-physician requirement posing some bureaucratic challenges, it has been described as functionally permissive given that there is broad legal interpretation of laws (31). Compared with the polarized environment of abortion care in the U.S., UK physicians view abortion as integrated into healthcare rather than a contested ideology. Its cultural acceptance and protection of both patients and physicians is further demonstrated by its adaptation to modern practices, with the “Pills by Post” Program which was made permanent post-COVID (32). These narratives across locations reveal how policy becomes a clinical variable, affecting how physicians conceptualize their role in the dramatically changing landscape.

### Intersecting barriers to access beyond legislation

Across settings, participants described intersecting barriers even when abortion is legal, highlighting that legality alone does not equate accessibility. Most common obstacles include distance of travel, costs associated with care, physician and appointment availability, and other aspects of healthcare systems infrastructure.

Distance of travel and cost of transportation emerged as major determinants of access, especially in more rural areas or those further from procedural sites. The Californian and UK accounts of patients traveling long distances illustrate how spatial inequities restrict care even in areas with progressive legislation (2). Similarly, while procedures are free in ROI, concentration of services in a few urban centers hinders access for those in rural regions (2). These findings reinforce that infrastructural changes must also accompany progressive policy to ensure equitable care.

Financial and institutional barriers compounded these challenges. For example, the absence of insurance coverage, cost of travel, childcare, and missed work all intersect with socioeconomic disparities, adding an additional psychological burden (33). Even in progressive states, system navigation issues and scheduling limitations, along with the growing patient demand place an institutional burden, delaying care (9). Physicians touched upon the difference in “medical care for pregnancy” and “medical care for abortion,” reflecting systemic stigma. Emotional and cultural barriers further added to difficulty accessing care. Shame and anticipation of judgment add to emotional distress for patients (33). Additionally, physician beliefs continue to shape care, often subtly.

Many barriers to care intersect and add a layer of complexity beyond the legality of access. Addressing barriers requires not only adequate policy, but also appropriate infrastructure, resources, and normalization to ensure equitable abortion care.

### Physician role in advocacy and implications for change

The need for advocacy across specialities beyond obstetrics and gynecology was emphasized by interviewees. Advocacy was viewed as an extension of patient care through its many modalities including public education, legislative action, or mentoring trainees.

The experiences of ROI and the UK highlight the benefits of legislative change towards more accessible abortion care, suggesting that similar reforms in the U.S. can ease current burdens. ROI's transition in the years following the 2018 repeal provide an example of how aligning legislation, education, and clinical implementation can rapidly expand access, reduce stigma, and foster cultural change. However, additional advocacy work can be done to address remaining barriers including the mandatory three-day waiting period, expanding access for those not near a city, expanding care to later-gestation abortions, and continuing to decriminalize abortions (9, 34). Similarly, within the UK's long standing abortion laws, there is an argument to be made to modernize and further decriminalize abortion as the logistical bureaucracy is still rooted in a conservative, Victorian era framework (35, 36). Together, these physician narratives and ongoing areas of progress highlight how the same medical procedure can exist in entirely different moral and logistical realities depending on ongoing changes in policy.

Based on physician perspectives and lessons from ROI and the UK, the following may serve as recommendations that U.S. states or health systems can adopt to progress abortion care. These include: (1) centering patient narratives to reframe abortion as healthcare rather than ideology; (2) implementing structured training and mentorship programs even in resource-limited environments; (3) addressing barriers arising from healthcare infrastructure; (4) fostering peer support systems such as digital communication networks; (5) empowering physicians as advocates and educators; and (6) pursuing state-level policy reforms that prioritize safety, continuity of care, and professional autonomy.

However, entrenched legal and cultural battles in restrictive states indicate that such changes will be gradual and contentious. States like Texas and Georgia have already experienced barriers and deteriorating care, evidenced by documented deaths as well as increased rates of maternal mortality (37–39). Regardless, the preparation and resilience of providers and allies, the adaptations to telehealth and new delivery models of abortion pills much like in the UK, as well as the ongoing work in increasing travel access and funding demonstrate the power of individuals in mitigating the overarching effects of law (40). Adaptation, activism, and compassionate aid as well as the community of resilience created in this process can continue to strengthen care regardless of restrictive policies (40).

### Addressing physician-facing stigma and dispelling misconceptions

These accounts show that stigma, while most often associated with locations with restrictive policies, is also pervasive across geographies, causing harm on personal, professional, and societal levels and materially contributes to delivery of care (41). Participants balance patient-centered care with their fears of judgement or harassment. The contrast between U.S. and UK physician experiences with public hostility highlights how policy and culture also directly influence physician safety and well-being. Even in a professional capacity, abortion is often viewed as a controversial service rather than standard healthcare, as evidenced by hidden conscientious objections and the discomfort of colleagues that further contribute to physician isolation and perpetuate misconceptions in care (42). However, abortion has been routinely shown to be a safe, routine procedure when performed appropriately to gestational age and by well-trained physicians for medical necessity (43). From 2013 to 2020, the U.S. case-fatality rate for legal induced abortion was 0.45 per 100,000 abortions, demonstrating its safety compared to childbirth and many other medical procedures (44). Clear, informed public education about abortion care, protection of providers from harassment, allyship, and appropriate language around care can dispel misconceptions and reframe abortion as essential healthcare, improving the environment for patients and providers alike.

### Contributions, strengths, and limitations

This study adds to the literature by offering a rare, cross-national, qualitative comparison of abortion-trained physicians’ experiences. Much of the existing research on abortion access has focused on patient outcomes or legal analyses. Our study complements this by foregrounding the voices of physicians and exploring how policy, culture, and training intersect to shape clinical practice. Importantly, we situate the post-Dobbs U.S. landscape within a global context, drawing attention to how other nations, specifically the ROI, have transitioned to increased access in care.

The strengths of this study include its international comparative design and the use of rich, first-person qualitative data that reveal both the structural and emotional dimensions of abortion care. By amplifying physician voices across diverse legal settings, we offer insights that can inform future policy, training, and advocacy strategies. However, limitations include the purposive sampling of physicians from exemplar regions, which causes geographic clustering of participants, and may not reflect the full range of experiences across each studied location or give context into abortion care in other countries. Additionally, while qualitative research allows for deep contextual understanding, these perspectives are drawn from a specific sample of abortion-trained physicians who self-selectively participated and may not represent the views of all clinicians in each region. Future work should include broader sampling across additional U.S. states and other countries, and explore patient perspectives in parallel to physician accounts.

## Conclusions

This study underscores how legal frameworks fundamentally and differentially shape abortion access, physician training, and patient care. Across California, Texas, the United Kingdom, and the Republic of Ireland, physicians were all committed to patient-centered care, but acted within their capacities shaped by policy. Barriers such as legislation, training limitations, geographical and financial limitations, regulatory challenges, and social stigma underscore the need for standardized training, better infrastructure, evidence-based policies, and physician advocacy to ensure accessible abortion services. The resilience of physicians in navigating fragmented systems and the need for coordinated responses that align training, access, and care are highlighted. The experiences and insights shared by interviewees emphasize the importance of medical autonomy, patient-centered care, and the need to dispel misconceptions about abortion. Ongoing challenges faced by physicians in regions with restrictive laws highlight the need for reforms to protect and support both physicians and patients in accessing safe and legal abortion services.

As abortion continues to be contested across countries, the experiences of physicians, both constrained and empowered by their legal contexts, offer insights into how health systems can either uphold or undermine reproductive autonomy. By contextualizing post-Dobbs America alongside countries that have transitioned from conservative to progressive care, this study emphasizes the role that reproductive policy plays in shaping access, clinical practice, patient outcomes, and honors patient autonomy. These observations urge us to consider the structures of training, how to best address systemic barriers to care, reflect on our own biases, and how to continue to advocate for patients.

## Data Availability

The raw data supporting the conclusions of this article will be made available by the authors, without undue reservation.

## Appendix 1

Interview Questions

1. How did you come to the decision to provide abortion services?
2. Describe how you received your abortion training. (Please highlight any support or opposition you received throughout training.) Were there any issues/opposition with getting this training?
3. Describe your knowledge of abortion laws where you practice.
4. Can you share an anonymous patient story that has impacted your practice?
5. How does policy inform/affect your clinical practice?
  - CA & TX: How does the Dobbs Decision inform/ affect your clinical practice?
  - UK: Ireland historically sent pregnant people to receive abortions in the UK. Do/how do Ireland's abortion laws affect your clinical practice? (Before and after the repeal of the 8th Amendment.)
  - ROI: How does the legalization of pregnancy termination inform your clinical practice?
6. How does social stigma impact your practice?
7. Have you ever felt conflicted morally or legally in your practice?
8. Do you fear for your safety?
9. What do you think are the greatest barriers for pregnant people to obtain abortions? *Prompt with fishbone headings* (Table 1)*.* Please choose the top 3 barriers. Discuss the changes (if any) you made to the fishbone diagram.
10. What steps does a pregnant person take if they want to obtain abortion services? What will the follow through look like on their end? How much will it cost?
11. What role do you feel physicians should play in guiding the direction of abortion regulations in the future?
12. What is a common misconception or piece of misinformation about abortion care/procedure that the general public tends to believe, that you wish you could rectify? What is something about abortion care/procedure that many people outside of the medical community don't know but should?
